# Malignant acanthosis nigricans: a case report

**DOI:** 10.1186/s12886-020-01708-2

**Published:** 2020-11-13

**Authors:** Xiaoyan Zhang, Rongjun Liu, Yiyun Liu, Shuang Zhang, Hong Qi

**Affiliations:** 1 Department of Ophthalmology, Peking University Third Hospital, 49 North Garden Rd., Haidian District, 100191 Beijing, PR China; 2 Department of Ophthalmology, Eye Hospital, China Academy of Chinese Medical Sciences, Beijing, PR China; 3 Department of Pathology, Peking University First Hospital, Beijing, PR China

**Keywords:** Dry eye disease, Gastric adenocarcinoma, Malignant acanthosis nigricans, Case report

## Abstract

**Background:**

To report a case of malignant acanthosis nigricans with two unusual aspects, including the patient’s young age and the development of filiform papillomas on the eyelid margins.

**Case presentation:**

A 30-year-old woman presented with dry eye symptoms. Examination revealed filiform papillomas on the eyelid margins, gums, lips, hands, and axillae and excessive pigmentation localized to the neck, axillae, and groin. Biopsies of stomach, pancreatic, and thyroid lesions revealed gastric adenocarcinoma, pancreatic adenocarcinoma, and thyroid cancer, respectively. Systemic investigations showed gastric adenocarcinoma with metastatic spread. The patient was ultimately diagnosed with malignant acanthosis nigricans and died 4 months later.

**Conclusions:**

Acanthosis nigricans on the eyelid margins with a velvety overgrowth is highly suggestive of an internal malignancy, and full systemic investigations are warranted in these cases. In this patient, early signs were ignored, leading to the loss of a timely diagnosis and treatment.

## Background

Acanthosis nigricans is rare. It was independently described by Pollitzer [1] and Janovsky [2] in 1890. This disorder is characterized by hyperkeratotic, pigmented lesions on the neck, face, groin, umbilicus, and axillae. Velvety overgrowth of the skin at the flexures may be accompanied by filiform growths around the lips, tongue and hands [3].

There are two forms of acanthosis nigricans, benign and malignant, with the underlying disease mechanisms differing between the two types. Benign acanthosis nigricans can be caused by genetic and endocrine factors, obesity, and certain medications leading to elevated insulin levels [4]. Malignant acanthosis nigricans, on the other hand, is considered to represent a paraneoplastic syndrome co-occurring with cancers and is associated with internal malignancies, particularly gastric adenocarcinomas (55–61%) [5].

Mucous membrane involvement is rare in acanthosis nigricans and is suggestive of the malignant form [6]. Here, we report the case of a patient presenting with bilateral filiform papillomas on the eyelid margins who was subsequently diagnosed with malignant acanthosis nigricans. Unlike benign acanthosis nigricans, malignant acanthosis nigricans has generally been reported to occur in older populations [7]. In this case report, however, the patient was only 30 years old.

## Case presentation

A 30-year-old woman presented to the ophthalmology department with a 3-month history of swollen eyelids margins and dryness of the eyes. She had previously been diagnosed with dry eye disease at other institutions and treated with artificial tears without improvement. The patient also described development of multiple verrucous papules on the dorsa of her hands starting 4 months prior to presentation, with a subsequent diagnosis of atopic dermatitis at the dermatology department. In addition, the patient noted lip swelling starting 3 months prior to presentation and excessive pigmentation of the neck, axillae, and groin starting 6 weeks prior to presentation. She also described a feeling of discomfort in the upper abdominal region and unexplained weight loss over the prior 3 months. The patient denied a family history of cancer, history of drug use, history of hoarseness, or a personal medical history of obesity, diabetes, or other endocrine disorders.

**Fig. 1:**
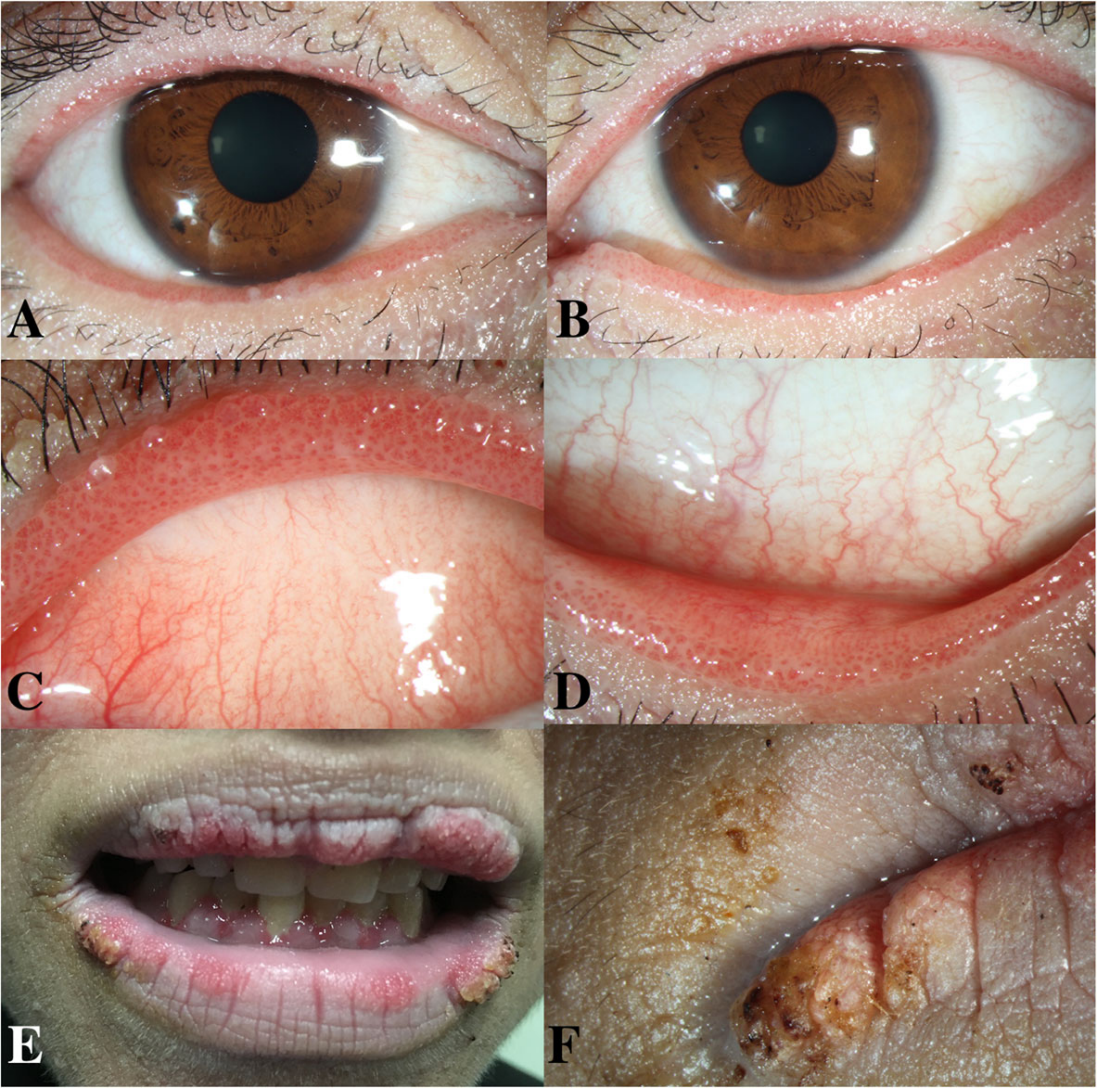
Patient images. **a** Anterior segment photograph of the right eye and **b** the left eye, demonstrating papillomatosis of the eyelid margins. **c** Magnified image of the upper eyelid margin of the left eye. **d** Magnified image of the lower eyelid margin of the left eye. **e** Images showing filiform papillomas of the gums and lips. **f** Magnified image of the lips

**Fig. 2:**
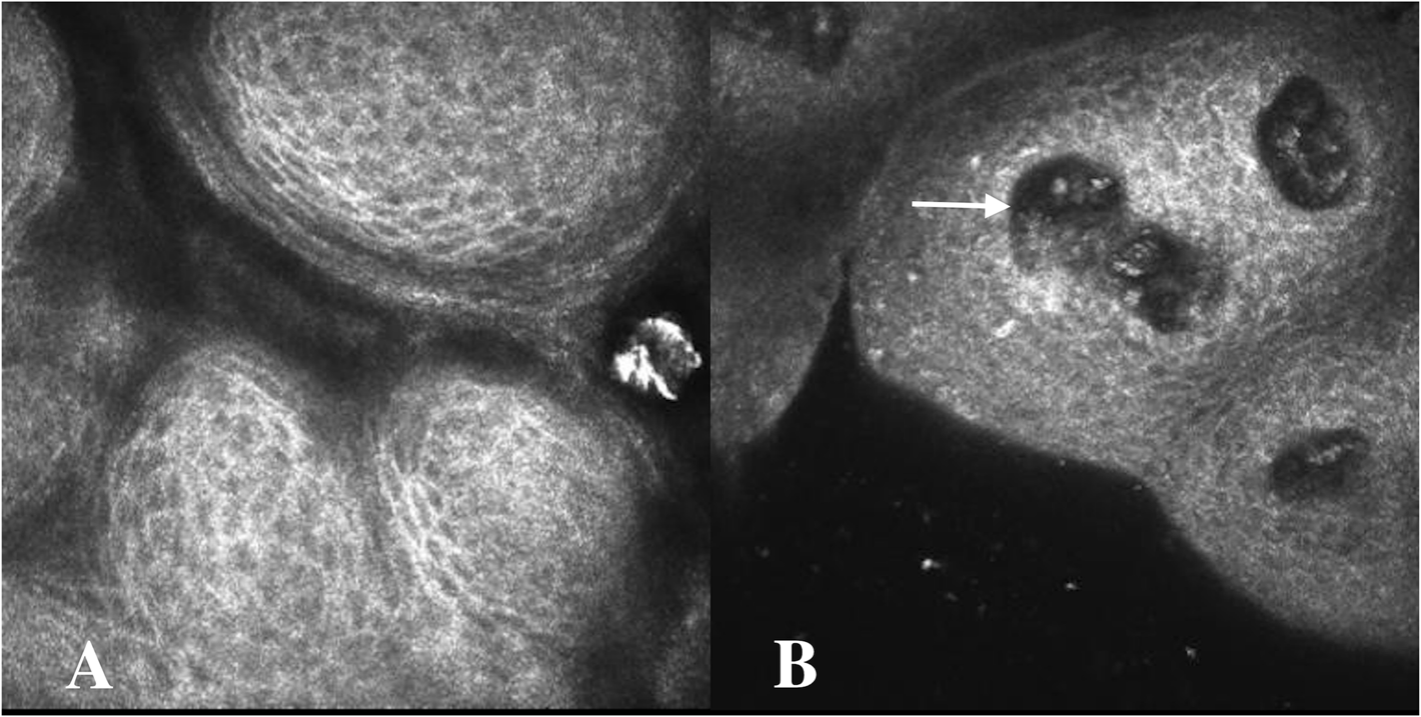
In vivo confocal microscopy images. **a**, Confocal microscopy of the eyelid margins showing conjunction cell proliferation. **b**, Confocal microscopy of the eyelid margins showing conjunction cell proliferation and central vascular structure (arrow)

Physical examination identified cervical lymphadenopathy; excessive pigmentation on the neck, axillae, and groin; and filiform papillomas on the eyelid margins (Fig. 1a-d), gums, lips (Fig. 1e-f), hands, and axillae. Corrected distance visual acuity was 20/20. The remainder of the anterior and posterior segment examinations were unremarkable. Tear break-up time was found to be 3 s. In vivo confocal microscopy images were obtained using the Heidelberg Retina Tomograph III/Rostock Corneal Module (Heidelberg Engineering, Dossenheim, Germany) confocal microscope. The images demonstrated conjunctival cell proliferation (Fig. 2a) and central vascular structure (Fig. 2b). Mean corpuscular volume levels (84.4 fL) were normal. Tumor marker evaluations were also performed, and carcinoembryonic antigen, carbohydrate antigen 19–9, carbohydrate antigen 125, squamous cell carcinoma-associated antigen, cytokeratin 19 fragment, neuron-specific enolase, carbohydrate antigen 72–4, tissue polypeptide antigen, carbohydrate antigen 242, and tumor supplied group of factors were positive. Ultrasonography revealed diffuse thyroid lesions and pancreatic hypoechoic nodules. Computed tomography imaging revealed left kidney and bilateral adrenal gland complex cystic-solid masses suggestive of neoplastic masses, with evidence of metastatic spread.

**Fig. 3:**
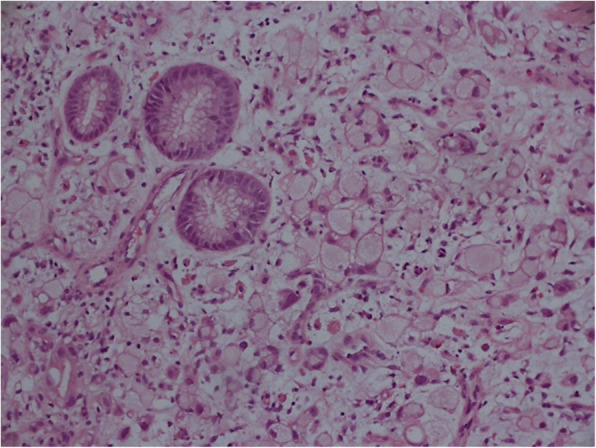
Biopsy results. The stomach section showing gastric mucosa with invasive adenocarcinoma with signet ring cell. Hematoxylin and Eosin stainning, original magnification × 200

The patient was treated with lubricant tear drops and topical steroids and referred to the oncology department. Lip and axillae biopsies revealed papillary structure. Stomach lesion was biopsies. The Haemotoxylin and Eosin staining was performed and the sections show gastric mucosa with invasive adenocarcinoma with signet ring cell (Fig. 3), glandular formation, and consistent with mixed-type gastric carcinoma by the Lauren classification system. Biopsies of the pancreatic and thyroid lesions revealed pancreatic adenocarcinoma and thyroid cancer. After these evaluations, the patient was subsequently diagnosed with malignant acanthosis nigricans associated with metastasis of gastric adenocarcinoma. Her condition deteriorated rapidly, and she died 4 months later due to cancer progression.

## Discussion and conclusions

Malignant acanthosis nigricans commonly occurs in older people, and involvement of the eyelid margins is unusual. The presence of acanthosis nigricans on the eyelid margins with a velvety overgrowth is highly suggestive of an internal malignancy. Here, we have reported an unusual case of a young woman with malignant acanthosis nigricans presenting with eyelid margin involvement.

In this patient’s age group, sarcoidosis, amyloidosis, lipoid proteinosis, secondary Sjogren’s syndrome leading to chronic keratoconjunctivitis sicca, severe allergic conjunctivitis with papillae, and benign acanthosis nigricans can present similarly and should be considered in the differential diagnosis. Sarcoidosis and amyloidosis can sometimes manifest with conjunctivitis with giant papillae. In our case report, however, the filiform papillomas were located on the eyelid margins, while the tarsal and bulbar conjunctivae were normal. The main symptoms of lipoid proteinosis are hoarseness from early infancy, yellowish papules over the eyelid margins and verrucous lesions on extensor surfaces (especially the elbows). In our case report, however, the patient had no hoarseness. The filiform papillomas appeared with a pink discoloration. And excessive pigmentation developed on the neck, axillae, and groin. In secondary Sjogren’s syndrome leading to chronic keratoconjunctivitis sicca, patients are often diagnosed with and treated for dry eye disease before the disease is suspected. Severe allergic conjunctivitis with papillae classically occurs in conjunction with conditions that have an underlying mechanism for hypersensitivity. The clinical manifestations of benign acanthosis nigricans are also difficult to distinguish from malignant acanthosis nigricans. However, in our case report, the patient had no relevant family or personal medical history suggestive of the benign form of the disease. In fact, the filiform papillomas on the eyelid margins were found to be associated with underlying malignant acanthosis nigricans in this patient, and the radiological investigations and hematological tumor marker tests showed evidence of advanced cancer.

Acanthosis nigricans with eye involvement was first noted in 1904 by Birch-Hirschfeld [8]. He described papillae at the eyelid margins and the tarsal conjunctiva, hypothesizing that the papillary hypertrophy on the tarsal conjunctiva occurred due to a chronic inflammatory response secondary to the eyelid margin changes [8]. In addition, Charlotte et al. [9] presented a case of a 65-year-old man with malignant acanthosis nigricans and an associated lung malignancy. He described papillomata at the eyelid margins, similar to our report.

In these patients, dryness of the eye may be caused by mechanical irritation resulting from the abnormalities of the lid surfaces. Lubricant tear drops and topical steroids can be helpful for management of these ocular symptoms [10]. The dermatologic manifestations of malignant acanthosis nigricans have been noted to improve or even resolve after surgical resection of the causative tumor; however, they may worsen again as metastases develop [9, 10].

The limitation of our study was the lack of histopathologic examination of the eyelid margins. In our case, in vivo confocal microscopy images demonstrated conjunctival cell proliferation. In previous case report, histopathologic examination revealed lid papilloma with well-differentiated epithelium in the conjunctiva [9]. It is useful for a timely diagnosis.

The mucosa and the skin are the organs of the body that can be most quickly and easily assessed. Acanthosis nigricans is a well-established marker of malignant tumors in the internal organs. In the present case of this 30-year-old woman without pernicious anemia, eyelid margin and skin lesions had developed more than 3 months prior to the diagnosis of gastric carcinoma and its metastatic spread, and full systemic investigations were warranted earlier in this patient’s clinical course. Unfortunately, the early signs of the underlying disease were ignored in this patient, leading to the loss of a timely diagnosis and treatment.

## Data Availability

All data generated or analyzed during this study are included in this published article.
